# Exploring Goal Flexibility and Mental Health in University Students: A Qualitative Approach

**DOI:** 10.1002/smi.70195

**Published:** 2026-06-24

**Authors:** Ngai Yan Mary Chun, Aaron Simpson, Nikos Ntoumanis, Claire Willis, Marina Milyavskaya, Timothy Budden

**Affiliations:** ^1^ College of Psychology Central Queensland University Rockhampton Australia; ^2^ School of Human Sciences (Exercise and Sport Science) University of Western Australia Rockhampton Australia; ^3^ The Kids Research Institute Australia Perth Western Australia, Australia; ^4^ Danish Centre for Motivation and Behaviour Science University of Southern Denmark Odense Denmark; ^5^ School of Sport Exercise and Rehabilitation Sciences University of Birmingham Birmingham UK; ^6^ Department of Psychology Carleton University Ottawa Ontario Canada

**Keywords:** goal adjustment, goal pursuit, interviews, mental wellbeing, reflexive thematic analysis

## Abstract

University students face obstacles while pursuing their goals, and these challenges risk undermining their mental health. Goal flexibility has been identified as beneficial for promoting well‐being and protecting mental health; however, the relationship between goal flexibility and mental health challenges remains underexplored. This study aims to understand how students engage in goal flexibility during periods of mental health challenges. We interviewed Australian university (*n* = 20) and university college (*n* = 1) students, comprising predominantly psychology students (81%), recruited through purposive and snowball sampling and analyzed data using reflexive thematic analysis. We identified two predominant perceptions of goal flexibility: (1) modifying the pathway to a long‐term goal; and (2) acceptance that not all goals are achievable and moving on. Participants described using three strategies to promote goal flexibility: (1) health and social behaviors; (2) contemplative practice; and (3) ‘practical’ strategies. Participants reported a range of mental health challenges—most commonly stress, anxiety, and depressive symptoms, alongside longer‐term diagnosed conditions (e.g., ADHD), and we identified different patterns in how these affected participants' capacity to engage in goal flexibility. Goal flexibility processes were in turn experienced as supportive of mental health, primarily by reinforcing personal agency, with participants describing specific processes (e.g.., goal revision, freezing, and reengagement) as helpful for the specific challenge(s) they were facing. Participants expressed concerns that being “too flexible” with goals could have negative mental health consequences and undermine agency. Future research should conduct more targeted studies examining goal flexibility among individuals experiencing specific mental health challenges. Additionally, research should include diverse populations, such as students on‐campus, from varied disciplines, ages, socioeconomic, and cultural backgrounds.

## 1 Introduction

University students commonly face mental health challenges such as depression, anxiety, and stress. For example, 27.7% of Australian students experience depression (Rickwood et al. 2016) and 23.7% experience anxiety (Francis‐Taylor et al. 2023)—consistent with global estimates (Tan et al. 2023). Emerging adults at university balance coursework with part‐time jobs, new social opportunities, and unfamiliar ‘adult’ responsibilities (Arnett et al. 2014), whereas mature‐age students balance academic studies with full‐time work, family responsibilities, or career transitions (Stone 2019). These competing demands can cause considerable stress (Bekkouche et al. 2022; Karyotaki et al. 2020), forcing students to adjust goals when circumstances change or goals become unattainable. Stress, if persistent, may result in cognitive overload, burnout, and chronic stress. Given that mental health challenges—such as depression, anxiety, and stress—may both stem from and interfere with students' ability to adapt their goals, understanding how students navigate goal pursuit during periods of psychological distress is critical to supporting their wellbeing and academic functioning. We use 'mental health challenges' throughout this study as a deliberately broad umbrella term encompassing transient stress responses, anxiety and depressive symptoms, and longer‐term diagnostically distinct conditions (e.g., bipolar disorder, attention‐deficit disorders, eating disorders). We treat these as related but distinguishable phenomena that are likely to relate to goal flexibility in different ways.

We adopt *goal pursuit* as the organising perspective for this issue. Stress, anxiety, depression, and affect arise when people encounter obstacles, threats, and disruptions to their goals (Carver and Scheier 1990; Higgins 1987). Constructs from adjacent literature (e.g., coping, self‐regulation, emotion regulation) are themselves forms of goal‐directed activity, not alternative frameworks for understanding it (Carver and Scheier 1998; DeYoung 2015). The relationship between goal pursuit and mental health is constitutive (DeYoung and Krueger 2018), as mental illbeing is characterised by goal dysfunction, including persistent discrepancies between current and desired states, dysregulated affective signals about goal progress, blocked or unattainable goals, and failures to update goals as circumstances change. The question is therefore not how students cope with mental health challenges, but how mental health challenges shape, and are shaped by, the dynamics of goal pursuit.

*Goal pursuit* refers to the process by which individuals acts in their environment to achieve their goals (Carver and Scheier 1998; Milyavskaya and Werner 2018); *goals* are cognitive representations of desired end states a person is committed to attaining or maintaining (Ecker and Moors, 2024; Milyavskaya and Werner 2025). In university, goals range from specific grades to aspirations (e.g., entering a career), and are organised within hierarchies: shorter‐term goals serve longer‐term goals that are ideally connected to core values (Carver and Scheier 1998; DeYoung and Tiberius 2023). Traditional goal pursuit research emphasises persistence as a key determinant of success (Brandstätter and Bernecker 2022 *).* Indeed, some degree of sustained effort is necessary for progress and attainment. However, this overlooks how individuals adapt when circumstances change, or goals become unattainable. In such cases, individuals benefit from *goal flexibility*. Drawing on cybernetic models of self‐regulation (DeYoung 2015; DeYoung and Krueger 2018), we define goal flexibility as change in goal pursuit, in terms of *goal adjustment* (the *what*), *strategy adjustment* (the *how*) and *interpretive adjustments* (the *why)*. This framework treats the constructs reviewed below as proposed types of change a person can make to their goal hierarchy. We argue such a consolidating structure is needed because existing constructs in the goal change literature overlap conceptually but are rarely distinguished from one another.

*Goal adjustment* (the what) encompasses different ways the goal itself can change, distinguished by what is altered and the manner of change (DeYoung 2015). *Goal disengagement* involves withdrawing commitment and effort from a goal (Scholer et al. 2024), and ranges from *goal abandonment* (i.e., total withdrawal of effort and commitment), and *goal shelving* (i.e., temporarily withdrawing effort but not commitment), to *goal switching* (i.e., temporarily re‐directing attention and effort between goals) (Mayer and Freund 2025a; Scholer et al. 2024). Alternatively, *goal revision* involves increasing or decreasing the difficulty of a goal (Gee et al. 2018; Scherbaum and Vancouver 2010). Besides goal disengagement, goal adjustment could also involve *goal re‐engagement*, this refers to ‘re‐engaging’ with goal pursuit by generating new, meaningful goals, following goal abandonment. Although some past research uses these terms to refer to general tendencies, here we focus on the actual actions.

However, in many circumstances, individuals consider it more appropriate to retain their original goal while modifying the approach or strategy employed to achieve it. Strategies are stable patterns of thought and behavior directed toward goals (DeYoung 2015). *Strategy adjustment* refers to adjusting concrete actions, intermediate subgoals (DeYoung 2015), or goal prioritisation preferences (Alister et al. 2024). Alternatively, *interpretive adjustments* involve change in a goal's value, or more fundamentally, one's values. For example, if a goal no longer serves a value or longer‐term goal, the goal's value diminishes (Brandstätter and Bernecker 2022; Carver and Scheier 1998). Similarly, infeasibility undermines goal value. In some cases, change occurs in terms of core values (DeYoung and Tiberius 2023). For example, epistemically and personally transformative experiences, such as completing a university degree, change what individuals value in unpredictable ways (Paul 2014).

Each category of the framework of goal flexibility provides a distinct kind of protection for mental health in the face of obstacles, and the proposed mechanisms differ across the three categories. Within *goal adjustments* (the *what*), goal disengagement is theorised to reduce stress and anxiety associated with chronic discrepancy signaling when a goal is unattainable, by removing the source of unresolved feedback (Brandstätter and Bernecker 2022; Carver and Scheier 1998). Goal re‐engagement reduces depressive symptoms and supports mental wellbeing by restoring the goal directed activity through which positive affect and meaning are typically regulated (Barlow et al. 2020; DeYoung and Tiberius 2023). Goal freezing supports mental wellbeing (Hubley and Scholer 2022) when transient, and this relationship is conditional: freezing is adaptive when no opportunities exist to pursue a goal, but becomes maladaptive if one ruminates on frozen goals, or crucially misses goal pursuit opportunities (Mayer and Freund 2025a). Goal switching enables responsive engagement with opportunities to advance goals as they arise, distinct from the resource costs of multitasking (Kim et al. 2023). Goal revision is adaptive if one's estimation of their skills is significantly discrepant from reality (Vancouver and Purl 2017). Strategy adjustment (the *how*) protect mental health primarily by preserving the positive effect of progressing toward focal goals, avoiding depressive risks of premature goal disengagement and stress associated with repeated failure (DeYoung and Krueger 2018). Interpretive adjustment (the *why*) operates at the level of values themselves: re‐evaluating one's values and goals may be acutely distressing but facilitate a sense of purpose and meaning in the long term (DeYoung and Krueger 2018). The potential protective function of any one goal flexibility process likely depends on which symptom is operative, whether the goal in question is unattainable, peripheral, or central to one's value structure, and whether the conditions for the operation of goal flexibility are present.

Most goal pursuit and mental health research focuses on goal adjustment—particularly disengagement and re‐engagement—typically treating it as a capacity/individual difference and using quantitative self‐report measures in correlational designs or laboratory settings (Barlow et al. 2020; Healy et al. 2014; Riddell et al. 2022; Verschuren and Douilliez 2022; Wrosch and Scheier 2020). Less attention has been given to the broader goal flexibility construct, or to the bi‐directional relationship of goal flexibility with mental health. Preliminary findings suggest that different mental health challenges are linked to structurally different disruptions in goal flexibility. For example, individuals with major depressive disorder disengage faster from unsolvable tasks than healthy controls (Koppe and Rothermund 2017; Wrosch et al. 2013). However, depression is also associated with difficulty re‐engaging with goals, limiting opportunities to access mental health benefits of goal pursuit (Dickson et al. 2016). Few studies examine how anxiety affects goal adjustment, but available evidence suggests anxious individuals adopt avoidance‐oriented goals—focussing on preventing negative outcomes—rather than approach‐oriented goals (Dickson 2006) which may indicate that anxiety operates predominantly at the strategy layer (re‐orienting how a goal is pursued) rather than at the goal layer itself. These tendencies may constrain adaptive goal flexibility. Stress is rarely studied as an outcome of goal‐flexibility processes in its own right; the broader cognitive‐narrowing literature suggests its primary effect is to constrict the regulatory window over which goal flexibility can be deliberated (Fredrickson 2004). Considered together, these literature imply that stress, anxiety, and depression are not interchangeable inputs to a single goal‐flexibility output but may map onto different families of the cybernetic taxonomy outlined above — a differentiation that has largely been left implicit. Existing research rarely explores how individuals *experience* goal flexibility during mental health challenges. Past intervention work has predominantly focused on approaches to facilitate goal disengagement (e.g., Riddell et al. 2023, 2022) but provides limited insight into to the inter‐relationship of goal flexibility processes and mental health. Quantitative studies often overlook the real‐life context surrounding the decisions students make as they adapt, revise, or abandon goals. A qualitative approach can fill this gap by uncovering how people interpret and navigate goal flexibility in the context of mental health challenges, offering deeper insight into its meaning and role in everyday life. Crucially, whether the proposed framework's distinctions among the *what*, *how*, and *why* of goal pursuit are recognisable in students' lived experiences of goal pursuit remains an open question.

### 1.1 The Current Study

When university students face mental health challenges, their ability to pursue and adapt personal goals can be shaped—or constrained—by their psychological state. This qualitative study explores how students engage with goal flexibility during periods of mental health challenges. In doing so, it aims to inform the design of interventions that support adaptive goal processes. We explore the following questions.

- How do university students experiencing mental health challenges engage in and experience goal flexibility?
- What supports their ability to engage in goal flexibility?
- What type of relations do they perceive between goal flexibility and mental health?
- What are their recommendations for the promotion of goal flexibility and mental health?

## 2 Methods

### 2.1 Philosophical Perspectives

We adopted an interpretivist perspective underpinned by ontological relativism and a subjectivist epistemology (Denzin & Lincoln, 2012). Ontological relativism recognizes the complexity of social reality and the existence of multiple lived realities. From a subjectivist epistemology, knowledge is co‐constructed through the interaction between interviewer and interviewee, shaped by broader social and cultural contexts. To enhance rigor, we engaged in ongoing reflexivity throughout the study (Smith and McGannon 2018), recognizing the researcher's role in co‐constructing findings (Finlay & Gough, 2003). This study was conducted in Australia with English‐speaking domestic students, including both participants and the interviewer. The interviewer, a mature female student of Asian heritage, held qualifications in psychological science, counseling, business management, and IT, and was completing her Honors thesis online. She had relevant lived experience as a university student and parent of two emerging adults, which supported empathic engagement during interviews. Although English is her second language, there were no significant communication barriers.

Reflexivity extended into analytic decision‐making. The lead author's position (notably, a culturally and linguistically diverse background) shaped what she heard as salient, particularly accounts of competing demands across study, family, and health, which may be over‐represented in the early coding. Her psychology training created a risk of reading participants' lay descriptions through theoretical vocabulary they did not use. The analytic orientation was also shaped by the primary supervisor, with whom the cybernetic taxonomy of goal flexibility (the *what*, *how*, and *why* of goal pursuit) originated, and who encouraged a more deductive analytic stance than is typical in reflexive thematic analysis. Because the broader programme of research is concerned with clarifying the architecture of goal flexibility as an organising construct, the analysis was directed towards what participants' accounts could reveal about the proposed *what/how/why* structure rather than treating that structure as something to be discovered inductively. The themes are therefore products of an interpretive stance that takes the cybernetic taxonomy as a working scaffold; a team without that prior commitment might reasonably have organized the same data differently.

A qualitative design was selected because the questions of interest concern processes that are constitutively contextual and processual: how students operate on their goal hierarchies under conditions of psychological challenge, and how they make sense of those operations. Existing quantitative work (e.g., Barlow et al. 2020; Wrosch et al. 2013) has primarily characterized goal flexibility in terms of goal adjustment, as a between‐person capacity assessed by self‐report. While valuable for establishing prevalence and broad correlates, capacity instruments cannot easily resolve the structural distinctions our research questions targeted—for example, whether a participant's reported difficulty “letting go” of a goal, broadly framed as goal disengagement in much of the extant literature, reflects prolonged or total abandonment, frozen commitment with preserved value, rumination, or a wholesale change of higher‐order values. Such distinctions, particularly in exploratory work, are well‐suited to qualitative designs grounded in lived accounts, where the operation performed on the goal can be interpreted within the broader context of participants' experiences.

### 2.2 Recruitment and Participants

Ethical approval was obtained from both the Central Queensland University Human Research Ethics Committee (HREC2024‐047) at the lead author's institution and the University of Western Australia Human Research Ethics Office (2024/ET001032). We used purposive and snowball sampling (Sparkes & Smith, 2013) to recruit university students from diverse backgrounds and courses. Inclusion criteria required participants to be 18 years or older and either currently enroled in, or recently graduated (within 12 months) from, an Australian undergraduate or postgraduate course. Participants completed a pre‐interview survey with demographic questions, screening items, informed consent, and the option to receive a summary of study findings. To minimise the risk of exacerbating psychological distress, the pre‐interview survey included screening questions assessing acute mental health symptoms. However, no participants were excluded on this basis. Recruitment channels included university learning management systems, student social media groups, email lists, and word of mouth. Of 35 individuals who expressed interest, 21 completed interviews. The remaining 14 were excluded due to incomplete surveys (*n* = 4), not scheduling interviews (*n* = 6), not attending interviews (*n* = 3), or being unable to verify university enrollment (*n* = 1).

The final sample comprised 21 students, including eight males (38%) and 13 females (62%), aged between 19 and 54 years (*M* = 35.5, SD = 11.5). Participants were drawn from eight Australian universities and one university college; 52% were enroled at the lead author's university. 17 participants (81%) were studying psychology, with the remaining four enroled in social work, midwifery, environmental science, and creative industries. Levels of study spanned undergraduate (38%), honors (i.e., research‐focused fourth year students, 33%), and postgraduate (29%) coursework, with most studying in blended online mode (81%) and the remainder on campus (19%). We acknowledge that this sample is dominated by psychology students at a single university, and that the resulting accounts may carry the conceptual familiarity of participants whose discipline overlaps with the constructs under study.

### 2.3 Procedure

After pre‐interview survey completion, participants received interview scheduling details, were provided with a basic lay description of goals, and were asked to reflect on their goals and experiences. All interviews were conducted via Zoom (Zoom Video Communications 2024). Verbal consent was recorded separately before beginning the interview. The interviewer followed a semi‐structured guide to ensure consistency while allowing flexibility to pursue emergent topics. Interviews lasted between 50 and 70 min. At the end, participants were thanked, provided with mental health resources, and entered into a gift voucher draw, randomly selected on conclusion of the study.

## 3 Data Collection

The interview guide was developed by the lead author (NC), primary supervisor (TB) and collaborators (AS, CW, NN) based on expertise in motivation science, goal pursuit, and mental health. Questions were organized into three sections. The first section solicited background information about participants' goals (i.e., “What are your current goals?”), obstacles (i.e., “Can you recall any specific obstacles or challenges when you pursuing these goals?”), mental health challenges (i.e., “Would you please be able to share some experiences you have with mental health challenges?”), and their strategies for managing these challenges (i.e., “How did you handle [the mentioned mental health challenge]?”). In the second section, we asked about their perspectives on goal flexibility (i.e., “What does goal flexibility mean to you?”) and their experiences related to goal flexibility (i.e., “How have you engaged in goal flexibility at university in the past?”). We then explored the relationship between goal flexibility and mental health challenges (i.e., “In what way do you think mental health challenges affect goal flexibility?” and “Based on your description of goal flexibility, how did this affect your mental health?”). Finally, in section three, we elicited participant recommendations for supporting students in relation to goals, goal flexibility, and mental challenges (i.e., “What kind of support do you wish had been available to you during the challenging times you described earlier?”, and “To help university students with their goals, goal flexibility, and mental health, what would it look like?”). Following interview completion, the interviewer reviewed the Zoom‐generated transcripts to ensure verbatim accuracy based on the audio recording. We did not aim for traditional data saturation, which is inconsistent with interpretivist paradigms and student project constraints (Braun and Clarke 2021c; O’Reilly and Parker 2013). Data collection ceased based on pragmatic notions of time constraints, and that we had elicited sufficient information to construct a comprehensive narrative addressing our research questions and aims (Braun and Clarke 2021c). Data collection occurred between November 2024 and January 2025.

### 3.1 Data Analysis

We adopted a reflexive thematic analysis approach, a flexible method suited to interpreting rich, nuanced qualitative data without requiring alignment to a specific theoretical framework or technical expertise (Braun and Clarke 2019, 2021a, 2021b). To support analytic claims about how goal‐flexibility processes varied across psychological challenges, we summarized participants' self‐reported mental health presentations as set out in Table 1, drawn from the pre‐interview screening item, the demographic survey, and participants' own elaboration during interview. These reflect participants' accounts rather than standardised diagnostic instruments. Given the prevalence of stress (*n* = 19), anxiety (*n* = 14), and depression (*n* = 10), we focused on overlapping profiles across these three. Our analysis combined inductive (data‐driven) and deductive (theory‐informed) approaches and was underpinned by ongoing reflexivity to ensure transparency throughout the research process. We used QualCoder 3.5 (Curtain 2023), an open‐source qualitative analysis tool, to generate codes; Microsoft Excel to organize, refine, and cluster codes; and NCH Software ClickCharts Professional 6.83 (Software 2024) to develop visual representations of themes to inform critical friend discussions (Smith and McGannon 2018). We followed Braun and Clarke's (2021b) six phases of reflexive thematic analysis, with adaptations to suit the project context. First, the lead author documented initial impressions after each interview and began the familiarization process by reviewing each Zoom‐generated transcript against the audio recording, editing transcripts to ensure accuracy. Second, during the coding phase, initial codes were identified using QualCoder by the lead author, and example codes were discussed in regular weekly meetings between the lead author, primary supervisor (TB), and collaborators (AS, CW). Third, in the initial theme development stage, all coded data were exported to Excel, where codes were refined and grouped into preliminary themes. Fourth, themes were developed and reviewed through the creation of visual diagrams using ClickCharts, which helped cluster themes into broader categories. Visual diagrams and corresponding theme descriptions and examples were distributed to the primary supervisor and collaborators for independent review and were subsequently discussed during a series of regular meetings over a 1‐month period. These discussions centered on plausibility and analytic clarity, and discrepancies were resolved through dialog and re‐grounding in the data. These diagrams were used and intended to provide a visual representation to form the basis of critical friend dialogs and to revise the structure and content of the thematic analysis results (Smith and McGannon 2018). Fifth, themes were further refined, defined, and re‐named through multiple iterations, including rechecking codes where needed, with feedback and critical discussion with co‐authors assisting in theme refinement. Finally, during the write‐up phase, the student researcher draughted the analysis and continued to refine themes as part of an ongoing reflexive process.

**TABLE 1:** Demographics and mental health challenges.

Participant's pseudonym (number)	Age range (Gender)	Level of education (subject)	Family	Work	Reported mental health challenges
Violet (P1)	26–35 (female)	Honors (psychological science)	Common law partner	Part‐time	Stress, anxiety, loneliness, depression
Mia (P2)	46–55 (female)	Honors (psychological science)	Married	Part‐time	Stress
Sophie (P3)^a^	18–25 (female)	PhD (creative industries)	Single	Part‐time	Attention‐deficit/hyperactivity disorder, autism spectrum disorder, anxiety, depression, stress
Phoebe (P4)^a^	36–45 (female)	Master (social work)	Married	Unemployed	Stress, anxiety, impostor syndrome, and depression
Stella (P5)	36–45 (female)	Master (psychological practice)	Married	Unemployed	Stress, anxiety
Donna (P6)	46–53 (female)	Honors (psychological science)	Single	Unemployed	Bipolar disorder, depression, and post‐traumatic stress disorder
Olivia (P7)^a^	26–35 (female)	Undergraduate (midwifery)	Married	Unemployed	Stress
Iris (P8)	36–45 (female)	Graduate diploma (psychology advanced)	Married	Unemployed	Stress, anxiety, and attention‐deficit/hyperactivity disorder
John (P9)	18–25 (male)	Honors (psychological science)	Single	Part‐time	Depression, social anxiety
Brian (P10)	26–35 (male)	Master (psychological practice)	Single	Part‐time	Stress, anxiety, depression
Nora (P11)	36–45 (female)	Honors (psychological science)	Married	Part‐time	Perfectionism, stress, anxiety
Peter (P12)	26–35 (male)	Undergraduate (psychological science)	Single	Unemployed	Borderline personality disorder, stress, depression
Rose (P13)	26–35 (female)	Undergraduate (psychological science)	Single	Part‐time	Stress, anxiety, depression
Thea (P14)	46–55 (female)	Honors (psychological science)	Single mother	Full‐time	Stress
Heidi (P15)	18–25 (female)	Undergraduate (psychological science)	Common law partner	Part‐time	Perfectionism, stress, anxiety
Ruth (P16)	46–55 (female)	Undergraduate (psychological science)	Single mother	Unemployed	Stress, depression
Henry (P17)^a^	18–25 (male)	Undergraduate (environmental science)	Single	Part‐time	Procrastination‐related stress, substance use
Nolan (P18)	46–55 (male)	Honors (psychological science)	Married	Full‐time	Stress, anxiety
Tom (P19)	18–25 (male)	Undergraduate (psychological science)	Single	Part‐time	Procrastination‐related stress and anxiety, social anxiety
Zac (P20)	26–35 (male)	Honors (psychological science)	Common law partner	Part‐time	Procrastination‐related stress and anxiety
Liam (P21)	26–35 (male)	Undergraduate (psychological science)	Married	Part‐time	Stress, anxiety, depression

^a^ Participant studies on‐campus.

## 4 Results

Interviews produced 437 pages of single‐spaced text. Basic demographic information and reported mental health challenges are presented in Table 1. Sample composition (sex, age, institution, course, level, and study mode) is reported in the Recruitment and Participants section. Participants described current goals, encompassing five domains: (1) *academic and career goals*; (2) *family‐related goals*; (3) *wellbeing and mental health goals*; (4) *health and fitness goals*; and (5) *personal goals (*e.g.*, hobbies)*. Participants reported six common obstacles and challenges: (1) *study‐related challenges*—pressure of meeting grade standards for career goals, or study‐habit issues (e.g., time management and procrastination); (2) *family responsibilities*—uncertainty associated with caring for children or ill family members; (3) *financial pressure*—upholding work commitments and ‘paying bills’; (4) *physical health*—in some cases due to study and other commitments; (5) *cultural and social challenges*—including challenges arising from significant life events; and (6) *mental health illnesses and neurodiversity*—including challenges related to pre‐existing diagnoses (e.g., attention‐deficit/hyperactivity disorder, post‐traumatic stress disorder, autism spectrum disorder). Most participants experienced stress (*n* = 19), and frequently reported depression (*n* = 10) and anxiety (*n* = 14). We identified nine themes, under four higher‐order categories reflecting: (1) university students' perspectives on goal flexibility; (2) strategies students employed as they engaged in goal flexibility; (3) the perceived relationship between goal flexibility and mental health; and (4) participants' recommendations for promoting goal flexibility and mental health.

### 4.1 Perceived Meaning of Goal Flexibility Based on Experiences

Participants struggled to provide precise definitions of goal flexibility, but their reflections revealed rich, experiential understandings of its meaning in practice, articulating how they engaged in goal flexibility processes. We identified two themes reflecting these meanings: (1) *Modifying the pathway to a long‐term goal* and (2) *Accepting that not all goals can be achieved and moving on*.

### 4.2 Modifying the Pathway to a Long‐Term Goal

Participants commonly described goal flexibility as the ability to adapt their approach while staying committed to valued, long‐term goals. More specifically, participants considered goal flexibility in terms of persistence, as a means to protect their goals by accommodating change at more immediate, short‐term, and concrete levels. Phoebe (P4) illustrated this perspective as follows: “I'm not willing to compromise on where I'm going, but I'm willing to compromise on how I get there.” For many participants, the goals to which students referred, which were typically academic goals, were fundamental to their personal values. We identified three sub‐themes reflecting modifying pathways to achieving long‐term goals: (1) adjusting short‐term goals to accommodate long‐term goals; (2) adjusting commitments to lower‐priority goals; and (3) adjusting timelines for goal attainment.

#### 4.2.1 Adjusting Short‐Term Goals to Accommodate Long‐Term Goals

Participants implicitly differentiated goals hierarchically, referring to ‘long‐term’ and ‘big’ goals decomposing into manageable ‘short term’ and ‘small’ goals. By flexibly “splitting [a big goal] into parts and some milestones”, Violet (P1) noted that this process “reduces the stress level, the anxiety”. For Iris (P8), adjusting ‘sub goals’ or ‘small goals’ “[is] around finding opportunities in spaces to start working” to employ her new skills before further study as a provisional psychologist. For Stella (P5), flexibly adjusting ‘short‐term goals’ meant “[she had] choices and options is really calming”. Recognising “multiple options” meant Stella identified multiple “pathways”, protecting higher‐order goals when “one door closes for a goal pathway”.

#### 4.2.2 Adjusting Commitments to Lower Priority Goals

Participants engaged in goal flexibility by reassessing goal importance and adjusting commitments—typically scaling down or reprioritising low priority goals to protect capacity for academic or career goals. Peter (P12) framed goal flexibility as “adjusting your routine, your settings, and any other outside factor that's stopping you from achieving a goal”. These adjustments affected daily effort and energy allocation. For example, Zac (P20) revised his gym routine when facing study demands: “[I] aim for 5 days a week… [I would] go 4 days or go 3 days that week…I've still partly achieved my goal”. Zac ‘softened’ success standards for peripheral goals to accommodate academic priorities. Iris (P8) intentionally deprioritised social engagements to preserve higher priorities: [My] personal goal was to really do this [study] in a way that wasn't going to impact all of my family, [if] we've had events come up, and I've had to say no to them.”

#### 4.2.3 Adjusting Timelines for Goal Attainment

For participants, goal flexibility also involved revising expectations for the timeframe of goals to align with changing life circumstances, energy, and wellbeing. Several participants described literally adjusting assignment due dates: for instance, Donna's (P6) “goals don't really change that much”, whereas goal flexibility was “being able to move the deadlines”. Phoebe (P4) expanded the timeframe, explaining the goal‐focused motivation behind part‐time university study: “That's me being flexible. I could have tried to do it full‐time, but I adjusted to fit my life around my studies.” This approach protected Phoebe's progress toward various competing goals.

### 4.3 Accepting That Not All Goals Can Be Achieved and Moving on

Some participants conceived of goal flexibility in terms of a difficult but meaningful, process of ‘letting’ go or abandoning unachievable goals, centered on recognising and *accepting* limitations and evolving circumstances. Rather than signaling failure, these acts of acceptance were framed as necessary for mental health and adjustment to life circumstances. Nolan (P18) framed goal flexibility as an “ability to change your goals quickly and to accept that you may not be able to achieve one goal”. Thea's (P14) account of adapting to disrupted plans during the COVID‐19 pandemic highlighted the emotional disruption from ambivalence to acceptance: “So kind of crossing the bridge of, that, first you feel ambivalence, and then later on, you're accepting [the situation] and then finding other solutions.” Phoebe (P4) offered that goal flexibility was not only “accepting […] whatever's thrown at you” but also being able to “shift your goal to fix sort of a new direction or a new point.” *Acceptance* functions as a form of agency across these accounts, deliberately recalibrating in the face of obstacles and challenges, and by enabling goal flexibility critically conserves resources, effort, and time, for new goals, instead of fixating on an unachievable goal.

### 4.4 Strategies Employed to Engage in Goal Flexibility

We identified three overarching strategies participants used to support goal flexibility: (1) Health and social behaviors—engaging in activities that benefit body and mind (i.e., rest, exercise, socializing); (2) Contemplative practice—using reflection and meditation to recalibrate or redirect goals; and (3) Practical strategies—taking a pragmatic approach through forward thinking, cognitive load management, and proactive prioritization.

### 4.5 Health and Social Behavior

#### 4.5.1 Rest

Many participants viewed rest as a way to temporarily disengage from mentally demanding goals, often through sleep. Mia (P2) recalled, “Sometimes, I was not able to go and study. Close the computer, and I went to bed … I needed to rest my body.” Violet (P1) saw rest as a reset: “If … you can't even focus because you're feeling so stressed [then] you have to … try and reset. Because if you don't, you're not going to get anywhere… taking a break isn't a bad thing.” Others described ‘active’ rest, such as switching to low‐effort activities. Heidi (P15) shared: “I like to play video games…[it's] so relieving […] I forget about university. I forget about all my mental health issues.” Liam (P21) described rest as active engagement in absorbing activities like “playing with the kids […] engrossing yourself and […] drawing, coloring with the girls.” Whether passive or active, rest offered pause from cognitively demanding goals.

#### 4.5.2 Exercise

Participants widely saw exercise as a tool to support goal flexibility, both by enabling temporary disengagement and enhancing overall wellbeing. Rather than competing with academic tasks, it was seen as essential for managing stress and restoring motivation and perspective. Nolan (P18) explained: “When those endorphins kick in, you start feeling good.” He described how running helped him detach: “Sometimes, you just get into a bubble… It can be a bit of a mindfulness.” For Stella (P5), exercise helped sustain academic performance by alleviating pressure: “Exercise to reduce stress is something that I tried to implement, and then found that obviously, it was working.” Exercise served Brian (P10) as a strategic interruption from academic overload: “My goal was to improve my health […] finding time to slow down”. In each case, exercise was described as a tactical, restorative act that helped participants regulate stress and return to their goals with greater focus and renewed perspective.

#### 4.5.3 Social Activities

Engaging in social activities was another strategy participants used to support goal flexibility—not by directly changing goals, but by helping them emotionally reset, manage distress, and restore the psychological resources needed to stay engaged. For many, social connection buffered the emotional toll of goal pursuit and renewed motivation. Ruth (P16) described how it helped her refocus: “Being connected with others and … having positive connections… gives you the strength to carry through, … less of dwelling to the negativity of life.” Social engagement was also seen as a way to break cycles of rumination, creating space to reassess and re‐approach goals. Mia (P2) noted: “[Stress] becomes easy, and you become lighter” when supported by others. Others found connection with family and friends helped maintain momentum. Brian (P10) shared: “Spending time with [my family and friends], talking, venting… I found that that was uplifting… it helps with the isolation… just motivated me.” Social interaction offered an emotional reset—reducing stress, clarifying perspective, and enabling participants to re‐engage with paused goals or persist through difficult ones. Rather than disengaging entirely, social support helped participants adjust goals and renew clarity.

### 4.6 Contemplative Practice

#### 4.6.1 Reflection

Reflection was a key strategy supporting flexible goal pursuit. Through self‐reflection, participants evaluated goals, challenges, and mental health from new perspectives, enabling pausing, disengagement, or intentional re‐engagement. Mia (P2) used a diary to guide decisions: “I'm asking myself, is that really what I want? How I want? Am I in the right way—is it the right decision to do right now?” For Heidi (P15), reflection signaled change was necessary, as she was approaching a critical threshold: “Self‐reflection helped me recognize the stage before rock bottom… You only have goals when you see self‐improvement, when you think something needs to change.” For Ruth (P16), reflection reframed a traumatic experience and enabled re‐engagement with university goals: “[It recalibrated] my thinking… instead of being all depressed and downhill about it… looking at the bigger picture… I had to reconfigure what I'd had in my mind.” Brian (P10) reflected to recognise emotions and feelings: “Am I more angry than usual? More tired than usual? … recognising my feelings and emotions, my energy level… a sign that I need to rest and not work so much.” Reflection enabled adaptive disengagement (e.g., goal abandonment, switching, or freezing) and intentional re‐engagement, helping participants stay responsive to shifting needs and capacities.

#### 4.6.2 Meditation

Participants also used meditation and related practices to calm intrusive thoughts and regain emotional balance, especially when overwhelmed. Techniques included mindfulness, prayer, grounding, and breathwork—used not just for general wellbeing but to support adaptive goal engagement. Nolan (P18) used meditation after night‐time caregiving: “A little bit of adrenaline happening […] hard to get back to sleep […] I used a meditation app to focus on breathing and mindfulness.” Olivia (P7) turned to prayer during stressful shifts: “Before the start of each shift… I would be praying… that God would give me peace and strength to get through.” Donna (P6) described grounding rituals: “Making myself a cup of tea… pouring the water in the kettle… just trying to stay present, instead of going into the future or the past.” These practices reduced emotional reactivity, maintained clarity, and helped participants stay aligned with their goals—even when those goals needed to be adjusted.

### 4.7 ‘Practical’ Strategies

Participants described a range of forward‐thinking, practical strategies that supported flexible goal pursuit. These included: (1) *Forward‐thinking with a positive mindset*; (2) *managing cognitive load*; and (3) *proactively adjusting goal priorities.*

#### 4.7.1 Forward‐Thinking With a Positive Mindset

Students shared how a future‐oriented mindset helped them stay motivated and adapt to challenges. Mia (P2), who described herself as “a positive person,” actively looked for “positive things—what could be done better and how I can benefit from [this challenge].” This mindset helped her reframe difficulties as growth opportunities, often by adjusting her path towards long‐term goals. Rose (P13) found motivation through curiosity: “I just get excited about learning new things and seeing where my path takes me.” Violet (P1) viewed her sacrifices through a long‐term lens: “I'm making the sacrifice now. But… [it's] for me later on in life … it's not [going to] last forever.” Thea (P14) echoed this belief: “I know that the sun's [going to] come up. I know that there'll be a different solution… it might not be what I want… but something will happen.” This mindset did not remove obstacles but helped students stay engaged—especially when goal switching, or freezing was necessary.

#### 4.7.2 Managing Cognitive Load

To avoid burnout, students used planning and scheduling to manage mental load. Instead of focussing narrowly on one task, they created space for rest and perspective‐taking—key components of flexible goal pursuit. Heidi (P15) shared: “I make sure that I have time for myself… and just that alone has changed so much for me, even though I haven't… done any less work or reshuffled work schedules.” Her account highlights how perceived load—not just workload—can affect wellbeing. Mia (P2) explained how structure eased pressure: “Schedule it—it will be easier… you don't have to think … it reduces the stress level, the anxiety … [add] some fun that's not related to your goals.” These strategies prevented overload and enabled flexibility in timelines and priorities.

#### 4.7.3 Proactively Adjusting Goal Priorities

Participants emphasized planning ahead and setting clear priorities, freezing or switching goals as needed. Nolan (P18) focused value‐based priorities: “What's really, really important is, can that be done now, can that be held off? … What's the main priority?” Stella (P5) visually mapped assignment deadlines to stay ahead: “If assignments came up… I would prioritize the study… I tried to be quite proactive… to do them earlier rather than leave them to the last minute.” Liam (P21) worked ahead to account for parenting duties: “Being on top of everything like I was a week early.” Mia (P2) linked prioritisation with clarity and motivation: “The more clear and more internal desire you have, the easier [it] will be to achieve this goal.” After finishing Honors, she deprioritized weight loss: “I don't find right now a really strong desire in me and motivation to lose weight, because my life is pretty busy, and I have another priority.” For her, prioritization meant flexible, values‐aligned decisions—such as adjusting goals to accommodate parenting and migrating.

### 4.8 Perceived Relation Between Goal Flexibility and Mental Health Challenges

Participants described a bi‐directional relation between mental health and goal flexibility, believing that mental health challenges impaired their ability to be flexible, and goal flexibility supported mental health. We identified two themes reflecting the impact of mental health on goal flexibility: (1) *Narrow and rigid mindset* and (2) *Low motivation*—and two themes reflecting how goal flexibility influences mental health: (3) *Positive mental health through goal flexibility and agency*; and (4) *When flexibility becomes aimlessness*.

### 4.9 Impact of Mental Health Challenges on Goal Flexibility

#### 4.9.1 Narrow and Rigid Mindset

Participants described psychological states linked to mental health challenges—especially stress and anxiety—as antithetical to goal flexibility. While few specified how symptoms impacted particular flexibility processes, they consistently portrayed these states as narrowing their perspective, limiting holistic evaluation of goals, and shortening the timeframes over which they could plan or act. These states often led to reactive and rigid approaches to goal pursuit. Rather than adaptively reframing goals, participants described being consumed by urgency or emotional overwhelm. Iris (P8), for example, noted that under stress and anxiety, she felt “ruled a lot by how [she was] feeling, rather than how [she] cognitively would assess a situation.” In these moments, she lost access to broader, more strategic thinking. This shift to reactive goal pursuit—focused on immediate problems—reduced opportunities to reassess long‐term goals, but enabled flexible disengagement from shorter‐term goals. Peter (P12) shared that a “poor state of mind” means “Drop everything in order to fix [the] immediate problem”, while John (P9) observed that “rigid” thinking, “if you're not [going to] be in the right mental health, then you're likely to make a lot of mistakes”. Violet (P1) explained how university amplified her underlying anxiety: “University has really brought out […an] anxious side of me … Just by having that constant stress of having something to do […] you're thinking, oh, have I forgotten to do something?” The cognitive load of juggling multiple goals left her in a constant state of tension and vigilance. Sophie (P3) described feeling overwhelmed and constrained, saying she felt “extreme,” “less flexible,” and “very threatened.” Together, these accounts illustrate how mental health challenges can trap individuals in an emotionally charged, rigid mindset—restricting their capacity to reflect, reprioritize, or step back, all essential for adaptive flexibility.

#### 4.9.2 Low Motivation

Participants described how low mood—particularly during depression or chronic stress—drained motivation needed to engage with or adjust goals. Instead of consciously revising goals some avoided them altogether, not by choice, but due to a lack of energy. Donna (P6) described: “I wouldn't even attempt to write an assignment because I know it'll just make things worse … like I can't achieve this goal … So just putting some things on the back burner and doing something easier.” Her low motivation not only blocked action but also prevented re‐evaluation. Ruth (P16) described the exhausting paradox of depression: “Too depressed to do anything at all … [they] make themselves sick trying.” Here, perceived obligation clashed with actual capacity, resulting in inaction and distress. Mia (P2) similarly explained that stress reduced both desire and drive: “When we're in stress, we can have low mood and we have lower desires, lower motivation, and we just don't want to do something.” These reflections reveal how low mood impairs not just pursuit, but the motivation needed to reflect, reprioritize, or redirect efforts. When this reserve is depleted, individuals can feel stuck—unable to abandon unworkable goals or initiate new ones.

### 4.10 Impact of Goal Flexibility on Mental Health

#### 4.10.1 Positive Mental Health Through Goal Flexibility and Agency

Many participants linked goal flexibility to positive mental health by affirming their sense of agency—the ability to make intentional choices, set priorities, and adjust course. Flexibility was experienced not as avoidance, but as an empowered response to life's demands. Stella (P5) described the calming effect of recognizing her autonomy: “It's a greater sense of control and autonomy … I've got choices and options.” Iris echoed the value of not feeling trapped: “You don't feel like you're backed into a corner. You feel in control … [and] less anxious.” Participants also connected flexibility to self‐compassion. Olivia (P7) shared: “I guess you are being kind to yourself. You'll be able to say, ‘Actually, I don't need to achieve that goal right now. It's better for my mental health to step away.’” Heidi (P15) described it as a dynamic balance, “like yin and yang,” enabling her to shift between socializing, study, and self‐care—and later re‐engage: “I turn that goal into something even better.” Across accounts, goal flexibility supported wellbeing by fostering a sense of control and intentionality, by choosing how, when, and why to pursue them.

#### 4.10.2 When Flexibility Becomes Aimlessness

Some participants cautioned that excessive or poorly understood goal flexibility could become counterproductive. Instead of fostering agency, it could erode purpose and direction, and foster disconnection, or aimlessness, and undermine their sense of progress. Stella (P5) reflected on the emotional and mental health cost of abandoning a valued goal:

An important goal … gives you a lot of a great sense of accomplishment when you reach it, but if something was to thwart it… there's a great sense of disappointment … that could really cause some mental health distress.

Zac (P20) warned that constantly changing goals strips them of meaning: “They're not really goals … if you move the goals all the time.” Peter (P12) shared that excessive flexibility in high school left him without a stable endpoint: “There was no end goal,” contributing to psychological disorientation. Violet (P1) described a period of aimlessness as feeling like she was “in the backseat of [her] own life,” reacting passively to circumstances. Together, these accounts present a converse view: when disconnected from clear values or direction, goal flexibility can lose its empowering function and become disorganising. Participants stressed that meaningful goal pursuit requires both adaptability and a sense of continuity—something to anchor to. Without this, goal flexibility risks becoming passive resignation rather than an active, intentional choice.

### 4.11 Recommendations

We identified two overarching themes for promoting goal flexibility and mental health: (1) Resource content and (2) Accessibility and continuity. Within resource content, participants proposed four key elements: defining goal flexibility, raising mental health awareness, offering strategies, and ensuring content is authentic and relatable. In terms of delivery, participants emphasised early and repeated delivery of content.

### 4.12 Resource Content

Participants provided insight into content perceived as instrumental to promoting adaptive goal flexibility and reducing mental health symptoms, including clear definitions, relations to mental health, career pathways, and delivering authentic and relatable content.

#### 4.12.1 Defining Goal Flexibility and Raising Awareness of the Relationship With Mental Health

Participants emphasised that despite benefits of goal flexibility, it remains poorly understood. As Henry (P17) remarked, “I feel like so many people are struggling [with goal flexibility], but they might not even know about it”, highlighting the need for clear, accessible definitions grounded in empirical research. Tom (P19) recommended intertwining definitions of goal flexibility with mental health: “The definition of goal flexibility would have to be there … the prevalence of mental health issues, .. And then kind of intertwine that to how that impacts goals and vice versa.” Participants saw mental health awareness as a precursor to goal flexibility, noting that unacknowledged distress often leads to rigid or avoidant patterns. Content could incorporate reflective questions—such as “Is [stress] impacting your health?” (Nora, P11), “Are you getting less than 4 h of sleep a night?” (Nora), or “Are you procrastinating every night feeling guilty … is it 12 o'clock at night?” (Heidi, P15)—as prompts to identify ineffective goal strategies. Peter (P12) suggested that understanding one's mental health needs enables more informed goal setting, and Stella (P5) emphasised the importance of recognising “maladaptiveness” in perfectionistic tendencies. Finally, Brian (P10) and Zac (P20) stressed that content should be culturally and developmentally appropriate. They also highlighted the need to balance promoting goal flexibility with caution about its potential downsides—specifically, how excessive flexibility might undermine sustained goal pursuit.

#### 4.12.2 Strategies for Study and Pathway‐Related Information

Participants emphasised that pairing knowledge of goal flexibility and mental health with practical strategies and clear career pathways is critical. Participants recommended including content on managing stress and academic demands, both of which shape students' capacity for goal flexibility. As Nora (P11) suggested, students would benefit from “toolkits on stress management […] strategies around stress, [identifying] signs you don't ignore.” Several participants highlighted the value of teaching concrete skills such as proactive planning and goal prioritisation to support more adaptive responses to stress and setbacks. Liam (P21) noted the importance of peer learning, suggesting students should hear from others who have faced similar challenges: “Actually just talking to other people that have been through those situations and what it's been like juggling, you know. Life demands—that would have been helpful.” Beyond stress management, participants called for clearer guidance on course planning and academic progression to help students adjust goals over time. Violet (P1) described early difficulties managing her workload and recommended that programs teach students “how to prioritize and manage your workload,” especially for those returning to study. She reflected on the lack of clarity in future pathways within her Psychology degree and how better guidance on career routes—like accreditation or research options—could have helped her engage more intentionally with her goals. “It was something that I found was quite a hazy area,” she explained. Looking back, she felt earlier clarity would have helped her better align her efforts: “I could have been doing […] volunteer work to help me with later on.” In sum, participants viewed practical guidance on stress, study management, and future pathways as scaffolding for engaging in goal flexibility.

#### 4.12.3 Authentic and Relatable

Participants emphasized effectively engaging students requires relatable content grounded in ‘real’ experiences. Given the diversity of student challenges, relevance is key to fostering reflection on goal flexibility and mental health. Heidi (P15) suggested developing content describing scenarios students can see themselves in: “Maybe have a little relatable introduction where they'd be like, ‘Oh, wait! That is me.’ ‘Wait! I'm doing that,’ you know, that sort of thing.” Thea (P14) suggested students are drawn to authentic, rather than ‘polished’ success stories: “We don't always just want to hear the success stories… we want to hear real stories.” Brian (P10) emphasized that content must also be trustworthy and evidence‐based: “I don't know goal setting, motivation and all of that. So, I think… just ensuring that it’s research driven. But also… contextually relevant.” Participants believed that combining credibility with relatable storytelling would better support students in recognizing unhelpful goal patterns and engaging in more flexible, adaptive strategies.

### 4.13 Accessibility and Continuity

Participants emphasized that goal flexibility and mental health should be central, ongoing components of university learning resources—not a one‐off discussion. Ruth (P16) proposed: “it's something that should be introduced in the welcome pack … before people get into their units”, whereas others, such as Heidi (P15) also argued for a recurring format: “It needs to be somewhat mandatory to force everyone to view it … Maybe twice a year would be good at least.” Stella (P5) similarly recommended reinforcing the content during critical stress points in the semester: “About a week 3 aspect of a university term, because … week 5 is usually when assignments are starting to trickle in … and then again in like [weeks] 9 to 10.” Across accounts, there was a consistent call for easy access and repeated reinforcement to support flexible goal engagement and student wellbeing.

In addition to educating students, participants stressed the role of university staff and policy in recognizing the impact of mental health on goal flexibility. Sophie (P3) reflected on how support from a tutor could have changed her experience: “Say a tutor had [said] … I was so rigid … [because] I have to achieve a 97 … let's like relook at that … Open my eyes, and I probably would have developed more skills about being strategic about what I do set as a goal for myself.” Students valued moments when staff helped reframe expectations and reduce pressure. Nora (P11), overwhelmed by weekly readings, was reassured by her coordinator: “As long as you focus on the unit content, you'll be fine”—which helped her redirect towards manageable, goal‐aligned action. Rose (P13) similarly appreciated the Student Advisory team's support: “They help you sort of plan out what units you're doing, what terms—that takes that pressure off.”

## 5 Discussion

**FIGURE 1:**
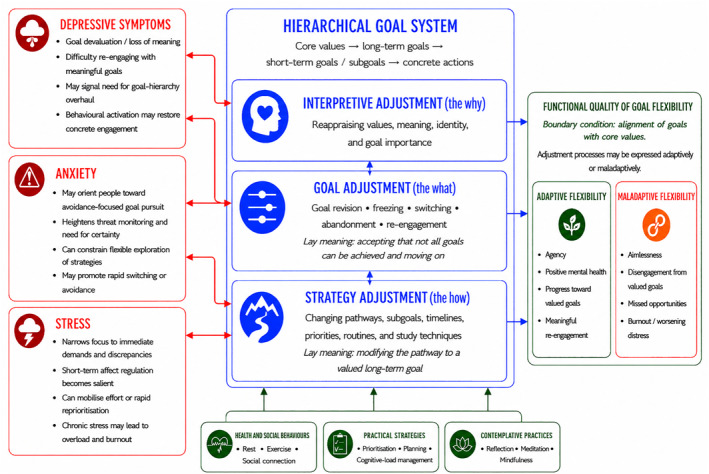
Tentative Integrative Framework of Goal Flexibility Processes and Mental Health Challenges. The figure presents a tentative integrative framework linking mental health challenges, hierarchical goal systems, goal flexibility processes, flexibility supportive strategies, and adaptive versus maladaptive expressions of flexibility. The hierarchical goal system includes core values, long‐term goals, short‐term goals/subgoals, and concrete actions. Goal flexibility is represented through interpretive adjustment (“the why”), goal adjustment (“the what”), and strategy adjustment (“the how”). Bidirectional arrows indicate proposed reciprocal links between depressive symptoms and interpretive/goal adjustment, anxiety and goal/strategy adjustment, and stress and goal/strategy adjustment. Health and social behaviors, practical strategies, and contemplative practices are shown as supports that may facilitate goal flexibility. Boundary condition shaping the adaptive versus maladaptive expression of goal flexibility is the integration of the revised goal system with core values. Arrows represent relations inferred from the qualitative analysis and relevant theory, not tested causal pathways.

This study explored how Australian university students experience goal flexibility while managing mental health challenges. Participants experienced goal flexibility predominantly in two ways: (1) adapting pathways to valued long‐term goals by adjustments in short‐term goals, commitments, or timelines; and (2) accepting the necessity of relinquishing unattainable goals and subsequently re‐engaging in alternative goal pursuits. Participants highlighted three clusters of strategies as promoting goal flexibility: health and social behaviors (e.g., rest, exercise, social engagement), contemplative practices (e.g., reflection, meditation), and practical strategies (e.g., proactive prioritization, cognitive load management). The analysis advances five interrelated contributions to the goal flexibility literature. First, participants' lay accounts provide preliminary support for the cybernetic distinctions between goal adjustment, strategy adjustment, and interpretive adjustment, lending experiential support to a novel taxonomy. Second, the two perceptions of goal flexibility identified—modifying the pathway to a goal and acceptance regarding goal unachievability—map onto strategy and goal adjustment, and participants articulated distinct psychological functions for each. Third, participants' framing of acceptance as a form of agency rather than resignation, contributes an experiential account of how adaptive goal disengagement is experienced. Fourth, participants articulated a boundary condition—flexibility without value‐anchoring becoming aimlessness—that specifies when goal flexibility itself may cease to be adaptive. Fifth, the strategy‐cluster of themes are best understood as means to facilitate specific goal flexibility processes, identifying a mechanism for existing coping strategies within a goal pursuit framing. These findings add qualitative depth to existing research by elucidating participants' lived experiences of goal flexibility processes and may inform the design of interventions to support students with mental health challenges by engaging in goal flexibility. To integrate these findings, Figure 1 presents a tentative conceptual framework linking mental health challenges, hierarchical goal systems, goal flexibility processes, supportive strategies, and adaptive versus maladaptive flexibility outcomes.

### 5.1 Theoretical Implications

We first consider what participants' accounts contribute to the cybernetic taxonomy of goal flexibility processes. The present analysis offers experiential evidence that these categories are psychologically meaningful to individuals experiencing mental health challenges as they pursue their goals. Consistent with hierarchical cybernetic theories (Carver and Scheier 1998; DeYoung 2015; Powers 1973) and contemporary multiple‐goal frameworks (Neal et al. 2017; Scholer et al. 2024), this study supports distinguishing three primary ‘families’ of goal flexibility processes: goal adjustment, strategy adjustment, and interpretive adjustment. Participants illustrated multiple goal, strategy, and interpretive adjustment subprocesses through their experiences. For instance, goal revision (Gee et al. 2018) occurred when participants negotiated assignment extensions or reduced secondary goal effort (e.g., gym sessions). Goal freezing (Hubley and Scholer 2022) was apparent when participants temporarily deprioritized goals during high demand periods (e.g., pausing social engagements during assessment periods). Goal switching (Scholer et al. 2024) involved shifting attention between different academic tasks based on urgency. Goal abandonment followed by re‐engagement was evident in mature‐age students describing career transitions into academic pursuits (Barlow et al. 2020). Notably, participants framed acceptance and the relinquishing of unattainable goals as forms of agency rather than resignation, and as a deliberate means to conserve resources for goal re‐engagement. This framing complements goal adjustment research, and offers a candidate explanation for why goal adjustment capacities reliably support improved mental wellbeing and illbeing (Barlow et al. 2020). Participants' accounts offered preliminary support for strategy adjustment as a distinct goal flexibility process in the face of mental health challenges. Participants adopted specific strategies—such as adjusting study techniques, proactive planning or managing cognitive load, and contemplative practices (e.g., reflection or meditation)—to stay present and focused, clarify how they might best pursue their goals, and maintain engagement with their academic and personal goals despite fluctuating psychological states. Finally, interpretive adjustment was evident in participants' descriptions of identity changes associated with university study, reshaping their perception of available opportunities and the importance of goals over time (DeYoung 2015). The strategy‐cluster themes (rest, exercise, social engagement, reflection, meditation, prioritisation) are not novel as coping or self‐regulation activities, and we do not claim them as such. Their analytic value here lies in how participants described employing them, as means of enacting specific goal flexibility processes. In particular, our analysis foregrounds these strategies as means to support temporary goal disengagement (i.e., goal shelving) (Mayer and Freund 2025a, 2025b) as a means to manage cognitive load during periods of demanding goal pursuit, opportunities for reflective goal re‐evaluation, relocation of capacity across competing goals, or restoration of the resources required for goal re‐engagement. Our analysis also suggests that the aforementioned strategy‐cluster may help facilitate experience of positive affective states supportive of the broadened thinking (Fredrickson 2004) required to generate new and personally congruent goals.

### 5.2 Developing an Adaptive–Maladaptive Goal Flexibility Framework

Our findings underscore the need to develop a precise, nuanced vocabulary of goal flexibility processes—so we can discern when it enhances wellbeing and mitigates illbeing, versus when goal flexibility drifts into aimlessness. In this study, we adopted a broad, transdiagnostic view to understanding goal flexibility and mental health challenges. Nonetheless, there is reason to view specific negative mental health symptoms, such as stress, anxiety, and depression, as indicators of specific problems arising in the context of goal pursuit. Preliminary patterns suggest stress and anxiety narrow participants' regulatory focus towards imminent discrepancies (e.g., deadlines) and short‐term affect regulation. This narrowing could be adaptive (Orehek et al. 2011)—mobilizing effort or prompting rapid goal switching—so long as individuals can periodically ‘zoom out’ to reassess superordinate goals and strategies (Fredrickson 2004). When narrowing becomes chronic and metacognitive reappraisal is blocked, it may become maladaptive, potentially resulting in tunnel vision, perseveration, and burnout.

In the context of goal pursuit, depressive symptoms appear to operate differently, and may reflect the absence of meaningful goals, or significant problems in one's goal hierarchy that require deep reflection, or analytical rumination (Sevcikova et al. 2020). Translating the analytical rumination hypothesis (i.e., depression facilitates withdrawal from distractions, and promotes prolonged analysis of complex problems), to the context of goal pursuit, goal disengagement may be an adaptive response to promote complex problem‐solving and may signal the need to overhaul an individual's goal hierarchy. However, an alternative treatment perspective for depression, behavioral activation, or engaging in concrete daily behaviors, may help restore one's engagement in goal pursuit and a sense of purpose. From a hierarchical goal perspective, a question remains whether reconfiguring goals during depression is best approached “top‐down”, or “bottom‐up”. In either case, the specific role of goal flexibility processes in ameliorating depressive symptoms and promoting goal pursuit remains to be elucidated. A robust theory must be developed, specifying (a) discrete goal flexibility processes, (b) contextual moderators (e.g., goal domains, type, time horizons, and opportunities for goal pursuit), and (c) personal moderators (e.g., personality trait, meta‐cognitive capacity, value clarity), that may determine when and whether goal flexibility processes become adaptive or maladaptive in the context of depression, or other mental health challenges. Participants articulated a boundary condition that the goal‐adjustment literature does not directly specify: flexibility was experienced as adaptive when anchored to higher‐order values or sustained valued commitments, but as disorganizing when goals were revised in the absence of such anchoring. This generates a testable proposition—that the protective effects of goal flexibility on mental health may be conditional on anchoring new goals to values. Employing precise terminology clarifies these dynamics and provides the scaffolding necessary to theorize goal flexibility with precision demanded by psychological science (Farrell and Lewandowsky 2018).

### 5.3 Practical Implications

We identify several tentative suggestions informed by participants' accounts for universities to test empirically, in order to translate these findings into practical goal pursuit support strategies, particularly regarding the frequency, depth, and content coverage of goal flexibility and mental health awareness. Participants recommended universities integrate psychoeducational modules into orientation and core units. These should clearly define goal flexibility subprocesses and skills. For instance, mental contrasting with implementation intentions is a technique that can facilitate the distinction between attainable and unattainable goals and hence inform decisions to persist or disengage (Ntoumanis and Sedikides 2018; Riddell et al. 2023). In addition, participant accounts provide support for the meta‐cognitive distinction between goal shelving and goal abandonment, which may be a useful focus for student facing materials. Participants highlighted the importance of relatable and authentic content, consistent with various on‐campus initiatives to promote student mental health through pairing with students with shared characteristics (e.g., gender, cultural background) (Beauchamp 2019; Jeftic et al. 2023). Universities could implement a dynamic, mental‐health‐informed goal support system, where academic staff and counselors conduct regular check‐ins to combine mood screening with structured reflections on students' goal hierarchies, but further empirical work is required to test this hypothesis with co‐designed approaches integrating student perspectives. This approach may assist in identifying early signs of stress‐related narrowing or depressive devaluation, enabling timely adjustments to goals or strategies, such as shifting priorities during peak deadlines. Opportunities exist to integrate these interventions into mobile‐ or web‐based applications, enhancing accessibility and real‐time support (Nahum‐Shani et al. 2015). A key aspect of this system would be the use of just‐in‐time adaptive interventions, which could test the dynamic effects of targeted strategies aimed at promoting specific goal flexibility processes during periods of acute distress. These interventions would allow for personalised, contextual responses helping students to adjust their goals, strategies, and priorities in real time. Such interventions could help test which specific processes are most effective in mitigating stress and maintaining engagement with goals during pressure periods. The recommendations above should be understood as design hypotheses worth testing rather than as evidence‐supported intervention prescriptions. The present study describes participants' reported experiences and stated preferences and does not establish that any of the recommended supports would, if implemented, produce the proposed effects. Programme and policy‐level claims—for example, that universities should mandate particular psychoeducational modules, or that the proposed support system could identify early signs of stress or depression‐related goal devaluation in time to intervene—are framed as candidate components for intervention development and evaluation rather than recommended practice.

### 5.4 Limitations

While this study offers novel insights into the lived experience of goal flexibility among university students, several limitations should be acknowledged. First, the scope of these findings is more circumscribed than the language of “university students” might suggest. The sample was dominated by psychology students (81%), and over half (52%) were enroled at the lead author's university; the sample also skewed towards mature‐age and online‐mode learners. While this limits generalisation to traditional‐aged, on‐campus cohorts, it may also be a strength for the present aims: mature‐age learners have likely navigated more cycles of goal pursuit, disruption, and adjustment, and may therefore have a richer experiential basis from which to articulate when goal flexibility supports or undermines wellbeing. Psychology students may have prior conceptual familiarity with constructs such as goal pursuit, mental health, and self‐regulation, and may have been more able than students in other disciplines to articulate goal pursuit experiences in the vocabulary the interview invited. The themes are therefore most safely read as accounts of how this particular group of students made sense of goal flexibility under conditions of psychological challenge, and we have tried to use language consistent with that scope throughout the discussion. Generalisation to undergraduate, on‐campus, or non‐psychology student populations should be treated as an empirical question rather than as an established finding. Second, because the interview structure explicitly asked participants to reflect on “goal flexibility”, some of what they articulated is, in part, a response to a researcher‐introduced construct. We mitigated this in two ways: by anchoring our analysis to participants' descriptions of operations on their goal structure rather than to their use of the term, and by privileging language that participants generated unprompted. Nevertheless, the data should be read as co‐constructed through the interview process, and the term “goal flexibility” should be understood as our analytic frame rather than a pre‐existing folk concept. Third, the cross‐sectional and retrospective nature of the study limits conclusions about the temporal unfolding or causality of these processes; longitudinal designs such as ecological momentary assessment would be better suited to capturing how goal flexibility processes shift across the course of a semester or depressive episode. Fourth, our symptom‐domain coding rests on the pre‐interview screening item and on participants' elaboration of their experiences, not on standardised diagnostic instruments; the cross‐domain patterns reported in the discussion are consistent with prior quantitative work (Barlow et al. 2020; Dickson et al. 2016; Robson et al. 2023) rather than effect size estimates from a powered sample. Fifth, while we have identified what each strategy theme appears to do to the goal structure in participants' accounts, the present design cannot directly establish causal relations between specific strategies and specific goal flexibility processes. Sixth, we did not explore in detail how cultural background (e.g., individualism—collectivism), age, or maturity relate to goal flexibility, despite indications in the data that these dimensions matter.

### 5.5 Future Research Directions

Firstly, advancing an adaptive—maladaptive goal flexibility framework requires systematic research that integrates existing models of goal flexibility, mental wellbeing, and illbeing. This research should clearly specify goal flexibility process definitions and operationalizations and identify their relations with specific mental wellbeing and illbeing constructs. Second, identifying the specific conditions that distinguish adaptive from maladaptive forms of goal flexibility, such as stress‐ and anxiety‐induced perspective narrowing, or depression‐related goal devaluation, is critical. e.g., research could identify specific multiple goal pursuit dilemmas as signals that specific types of goal flexibility are adaptive, and ecological momentary assessment are attractive options for examining how goal flexibility processes unfold in real time and would help identify these ‘at‐risk’ moments during goal pursuit that would suggest specific goal flexibility processes would be adaptive. Thirdly, intervention studies are needed to test theories and evaluate mechanisms that enable goal flexibility processes. For instance, it is necessary to develop and test theories specifying when interventions targeting goal adjustment (e.g., goal revision or goal disengagement), strategy adjustment (e.g., identifying more effective ways to prioritise multiple goals), or value re‐evaluation would be most appropriate. To refine intervention design, it is essential to evaluate how specific strategies effectively target distinct goal flexibility processes, moving outcomes towards dynamic changes in goal pursuit and mental health. Fourth, future research should examine goal flexibility processes among clinical populations in which the structural distinctions described here could be tested with diagnostically defined samples.

## 6 Conclusions

This study offers novel insights into how university students navigate goal flexibility amidst mental health challenges. Students adapt their pathways towards valued goals and recognise when to abandon unattainable objectives, using strategies such as health and social behaviors, contemplative practices, and practical planning. The findings highlight a bidirectional relationship: mental health challenges hinder goal flexibility, while effective flexibility supports mental health by enhancing agency. Conceptually, the study lends experiential support to the cybernetic distinction between strategy and goal adjustment, reframes acceptance as a form of agency rather than resignation, and identifies value‐anchoring as a candidate boundary condition for adaptive goal flexibility. Recommendations call for continuous, authentic psychoeducational interventions within university curricula to help students manage goal pursuits in response to mental health challenges.

## Conflicts of Interest

The authors declare no conflicts of interest.

## Data Availability

The data that support the findings of this study are available from the corresponding author upon reasonable request.
